# Open Access: A PLoS for Education

**DOI:** 10.1371/journal.pbio.0020145

**Published:** 2004-05-11

**Authors:** A. Malcolm Campbell

## Abstract

Teaching -- and testing -- students creatively is a challenge, but new public databases and more accessible literature are now helping to develop the critical thinking skills of students

The next generation of life scientists are currently undergraduates—and the success of this generation depends upon the quality of the education they receive. It is clear the expectations for undergraduate education are changing (Collins et al. 2003). When the National Research Council published its recommendations for changing the undergraduate training of future life scientists, the BIO2010 report, access to student-based research was a primary recommendation: “Colleges and universities should provide all students with opportunities to become engaged in research …” (National Research Council 2003). As every investigator knows, research begins in the literature, not in the laboratory. Therefore, an unstated assumption of the BIO2010 report was that students need to have unencumbered access to the research literature in order to engage in research and become scientific leaders in the 21st century.

Early in my teaching career, I discussed graduate student preparation with a colleague at MIT. He said new graduate students knew about the different methods, they could even recite fine definitions—but if you asked them which method would be best to answer a particular question, they were uncertain. This reinforced my attitude towards teaching and testing. I realized that teaching science to students should be modeled on the way all scientists learn new information: in the context of an interesting question and on a need-to-know basis. This new style of teaching, “applied education,” would require me to reorganize reading materials for students, since most textbooks are written by someone who already knows all the information and has organized it accordingly. For example, describing membrane structure, protein structure, and signal transduction in Chapters 5, 12, and 15, respectively (spanning 227 pages) is not helpful for most students. It makes more sense to cover these three topics in close succession.

Gradually, I converted all my courses over to this “applied education” format in which students were learning new information the same way all other scientists do. I began by asking questions that could be answered by learning the information provided by textbooks or the literature. With time, I realized that published research papers are ideal teaching tools because they cover information in the context of an interesting question and new material is presented as needed. This led me to collect series of related papers to create my own course materials (see www.bio.davidson.edu/courses/Molbio/Publicschedule.html#anchor99574051). So, for example, in my classes students first read the elegant paper by Munro and Pelham (1987) that uncovered the tetrapeptide lysine–aspartic acid–glutamic acid–leucine (KDEL) retention signal for proteins destined to remain in the endoplasmic reticulum lumen. Then, students read four additional papers, one of which is composed of weak data and overinterpreted analysis. Through this series of papers, students learn to trust their own assessment of the data rather than the authors': this is a very substantial improvement in student thinking and in their attitude towards the literature. I do not emphasize the particular details of these paper, but I do want the students to gain higher-order thinking skills. Therefore, my tests consist of figures from research papers that the students have never seen before. They are asked to interpret the figures as they appear in the papers and/or to design new experiments to answer a new question, given what they have learned from the published figure. Testing them in this way, students very quickly understand that memorizing details is not productive, but learning how to read scientific literature and design well-controlled experiments is much more rewarding (see www.bio.davidson.edu/courses/Molbio/molecular.html#2003exams). Based on this success, I have designed my genomics course on the “applied education” principle (see below; see also www.bio.davidson.edu/genomics).

## Access to Information Changes Education

When I was a graduate student (in the late 1980s and early 1990s), PubMed was restricted to those institutions that could afford the subscription fee; now PubMed is freely available to all who have Internet access. This change in access to PubMed has significantly improved undergraduate training by providing students with the opportunities to do literature searches for their lab reports, papers, seminars, and of course original research. Free access to information in the life sciences has continued to evolve with the newest phenomenon in publishing—open-access journals. PubMed Central (http://www.pubmedcentral.nih.gov/) is a rich repository of and portal to open access articles, BioMed Central (http://www.biomedcentral.com/) publishes a growing number of open-access journals, and there are a few new open-access education journals such as *Cell Biology Education* (http://www.cellbioed.org) and the *Journal of Undergraduate Neuroscience Education* (http://www.funjournal.org). As the newest player in the open-access arena, *PLoS Biology* has further enriched the growing espritdes-corps of publishing and has already improved undergraduate education. My students now have equal access to a growing portion of the literature that Nobel laureates and investigators at wealthy institutions enjoy.

Interestingly, the push towards open access has led many subscription-based journals to permit “free access” two to 12 months after publication. These time-delayed free-access journals are helpful for course adjustments in the subsequent academic year, but not the current semester. Unfortunately, owing to the high cost of subscriptions for many journals, the library at my institution (like many other libraries) is forced to make difficult choices about which journals we can afford. The number of journal subscriptions goes down in proportion to the rise of subscription costs, but fortunately this loss is being offset by the creation of new open-access journals.

## The Promise of the Internet

**Figure 1:**
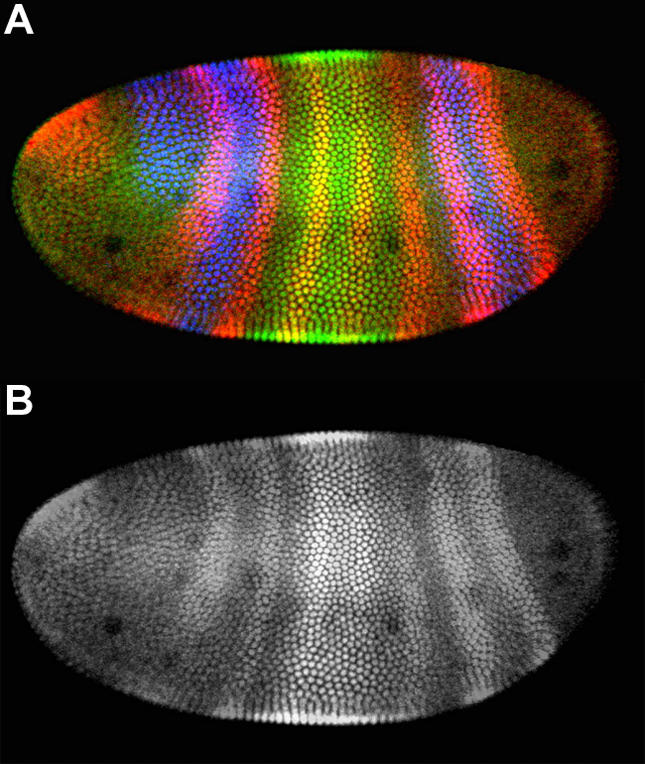
Comparison of Published and Photocopied Figures Example of an image that, when seen in color (A), is rich with information; much of this information is lost when it is photocopied by students (B), as when the original is held on reserve in the library, as is required for subscription-based journals, or is provided via interlibrary loan. This image of a developing fly embryo was labeled to reveal bands of differentially expressed proteins, with HAIRY in red, KRüPPEL in green, and GIANT in blue. (Image courtesy of Stephen W. Paddock, Jim Langeland, and Sean Carroll at the University of Wisconsin–Madison.)

I have been teaching undergraduates since 1993 and have noticed a trend in the way I teach—increasingly, I have provided research papers to my students so they can learn to read those papers and improve their critical thinking skills. One reason for my increased use of research papers is the development of PDFs. When I first started using journal articles in my molecular biology course, the class had to meet in the library so we could pass around the bulky bound volumes to detect the important subtleties often lost in photocopied versions of figures. Later, I learned how to scan the figures and generate Web pages so that I could project the images in class and so that students could print laser-quality versions of papers (see http://www.bio.davidson.edu/molecular). Now I use PDF files for students to print and for me to display in class with no loss of information due to reformatting or resolution problems (Figure 1).

With my increased confidence from using research papers in my molecular biology class, I began experimenting with research papers for my introductory students. First-year students are not ready to critically evaluate complex data, but they are beginning their first forays into reading review articles and occasionally original research papers. When introductory students make presentations of their findings in laboratory courses, increasing numbers are utilizing PubMed and PDF reprints when they are available.

Students have been reading primary research papers since well before PDF files became available, but the increased access to papers online and the improved quality of the format has significantly enhanced the use of research and review papers in the undergraduate curriculum. It is common for students in upper-level lecture and lab courses to read papers (DebBurman 2002; Hall and Harrington 2003; Kitchen et al. 2003; Mulnix 2003), and seminar courses are usually dominated by student presentations of literature (Wright and Boggs 2002; Hales 2003; Lom 2003).

It is worth noting that most colleges and universities are being told to reduce expenditures, and one frequent target of money-saving measures is the ever-increasing costs of library journal subscriptions. This fiscal reality will erode the pedagogical gains made by faculty who are already meeting one of the goals of the BIO2010 report by immersing students in the research literature. However, open-access journals are proving to be virtual oases in a desert of pay-per-view journals that are available on a sliding scale that favors the richest and biggest institutions.

## Using Open-Access Resources for Creative Teaching …

During the past three years, I have taught an undergraduate course in genomics (www.bio.davidson.edu/genomics) in which I capitalize on a confluence of two trends in the field: public domain databases and open-access journals (Campbell 2003). In my genomics class, students have three assignments for which they are required to mine databases for sequence, transcriptome, and proteome information (see www.bio.davidson.edu/courses/genomics/2003/cain/home.html). But genomics courses are not the only beneficiaries, since other classes at many institutions (e.g., introductory biology, biochemistry, cell development, genetics, microbiology, molecular biology [see http://www.bio.davidson.edu/courses/Molbio/standardsHP.html#anchor78181983], and neuroscience) require students to mine public domain databases (Dyer and LeBlanc 2002; Honts 2003). This year, we introduced genome database searching to our introductory biology students (see www.bio.davidson.edu/people/macampbell/Hope/DQ/DQ9.html and www.bio.davidson.edu/people/macampbell/Hope/DQ/DQ10.html). First-year students use Genome Browser and BLAST to determine the molecular causes of cystic fibrosis and Huntington disease, respectively. The benefit of public databases and open-access literature to educators is obvious and immediate. Images can be used in lectures, and papers can be distributed easily and on short notice for class use. There is no need to worry about limited access due to subscription costs nor an obligation to obtain copyright permission from publishers, which is a bothersome and sometimes expensive process for busy faculty members. By reducing nonproductive busy work for faculty, open-access journals have already created an environment that is improving undergraduate education today with long-term benefits in creating research-ready graduate students.

Students who are exposed to publicly available literature through their coursework often develop an expectation that all research papers will be freely available to them from any computer and become frustrated if they do not have access to all the journal articles they want and need to read. Increasingly, I have students sending me PDF files of open-access journal articles they have read and want to share with me. Who would have guessed that free access to journals would result in students mining the literature for relevant papers and sending them to their instructors for consideration? In addition to papers related to their own classes and research, students also enjoy learning about “hot topics” from scientific publications and those stories that quickly reach the popular press. Examples include the use of DNA microarrays and sequencing to identify the causative agent for SARS (Wang et al. 2003) and a good review article of small inhibitory RNA (Dillin 2003).

Two common educational goals are to encourage students to become skeptical of unsubstantiated claims and to enable students to evaluate data critically. One way to accomplish these goals is to capitalize on the natural curiosity of students and ask them to compare topics in the popular press to that in the scientific literature (see http://www.bio.davidson.edu/courses/genomics/2003/poulton/p21.html). Open-access journals make these two educational goals much more feasible because students can utilize current findings immediately without having to wait for interlibrary loans, which can take up to two weeks, can cost up to $20 per article, and can result in poor-quality black-and-white photocopies.

## … and for Thought-Provoking Testing

**Figure 2:**
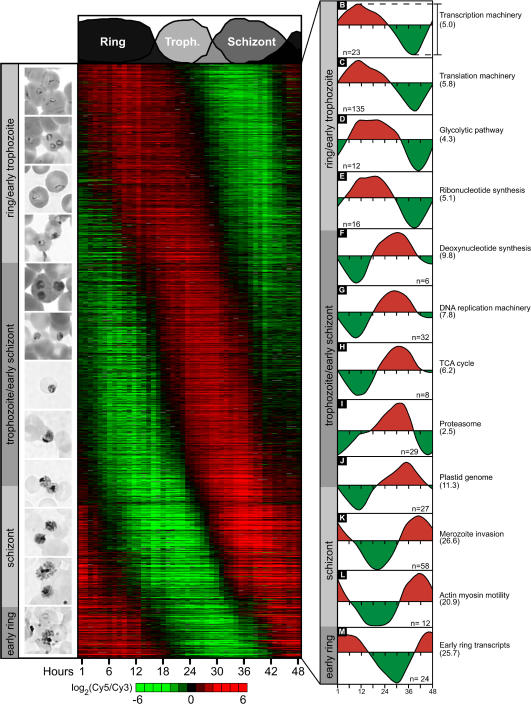
Example of Student's Data Mining for Exam Question Figure 2 from Bozdech et al. (2003) showing the gene expression profiles for 12 groups of genes expressed at different stages of *Plasmodium* life cycle inside red blood cells. Genomics students were asked to summarize this figure as a part of a take-home exam.

If we want students to achieve higher levels of thinking (Bloom et al. 1956), we need to model our courses so students can learn by examples and are rewarded for learning to critically evaluate data and for inspecting evidence before believing claims made by authors (Brill and Yarden 2003). Students quickly figure out what intellectual behaviors are rewarded in exams. If exam questions simply require students to regurgitate factoids, then higher levels of thinking are unlikely to be demonstrated by students. It is difficult to create good exam questions that cover the course material and reward students who have learned to read critically and to interpret data. Over the last few years, increasingly I have turned to current literature to find raw data for my exam questions. For example, for my genomics class in Fall 2003, I used a paper published in *PLoS Biology* that utilized DNA microarrays to analyze the life cycle of malaria-causing *Plasmodium* (Bozdech et al. 2003). I asked students to interpret several figures, using their own words (Figure 2). Owing to my choosing to use an open-access journal, my students also had full access to the supporting information, which two students utilized to enhance their answers. For this question, these two produced answers that were better than mine. Another exam question required students to mine a database associated with the Bozdech paper (see http://malaria.ucsf.edu/index.php). Students were asked to combine what they learned from the paper and the course and choose new proteins (in addition to the ones described in Bozdech et al. [2003]) that would make good candidates for vaccines based on the timing of gene transcription. In order to answer this question, students performed the first steps in real research, which rewards students for learning higher-order thinking skills.

At the end of their exam, students were given an opportunity for extra credit points (a maximum of three points out of 100 available on the exam) if they provided constructive criticism directly to the database curators. About 70% of the students sent comments, including this one: “In recently using your database, I found it difficult to search the *Plasmodium* gene expression data with multiple constraints. For example, it would be helpful if there were a way to identify all the genes within a certain functional group that fell within certain time or amplitude constraints. Is this possible in this database?” The curators very professionally responded to the students' suggestions, which resulted in three new search capacities being added to the database, as can be seen on the left side of the main page (see http://malaria.ucsf.edu/index.php). As a result of these professional interactions, students became participants in a community of scholars, interacting with investigators at the University of California, San Francisco, while taking their exams.

The use of open-access journals for teaching and testing has already improved my courses. I can provide exam questions that are more interesting, more educational, and more current. Furthermore, I accomplish two tasks simultaneously: I keep abreast of new developments in my field and I write exam questions. But what do students think? While I have not formally assessed student attitudes, I have collected information from end-of-semester course evaluations, including the following comments: “One of the best parts of the entire course for me were the exams. The exams really gave me an opportunity to show how I could work through real problems. This class definitely increased my critical thinking skills. Each test presented me with new ideas and problems to work through. I enjoyed the idea that each exam would be a learning experience.”

## The Future

Teaching is a lot like raising children. Like parents, teachers provide learning opportunities in part by modeling the behavior we want our students to learn. By choosing the most current literature as testing material, my students realize that I read the literature to stay current in my field and that there are always new opportunities to learn, analyze, and design experiments, etc. By my choosing open-access papers such as those published in *PLoS Biology*, my students benefit from free access to published research results. Free access to research literature enhances student learning and helps produce the next generation of graduate students, who are then better trained. Open-access publishing provides the right mix of benefits for educators and students alike.
